# Rest-Activity Rhythms With Cognitive Impairment and Alzheimer’s Disease (AD)-Related Plasma Biomarkers

**DOI:** 10.1093/geroni/igaf122.4185

**Published:** 2025-12-31

**Authors:** Ahmed Danquah, Ann Cohen, Beth Snitz, Yurun Cai

**Affiliations:** University of Pittsburgh School of Nursing, Pittsburgh, Pennsylvania, United States; University of Pittsburgh, Pittsburgh, Pennsylvania, United States; University of Pittsburgh, Pittsburgh, Pennsylvania, United States; University of Pittsburgh, Pittsburgh, Pennsylvania, United States

## Abstract

Rest-activity rhythms (RAR) may serve as early indicators of cognitive decline, yet their associations with cognitive impairment and Alzheimer’s disease (AD) biomarkers remain unclear. Thus, our study aims to examine the associations between objectively measured rest-activity rhythms, diagnosis of mild cognitive impairment (MCI) and AD, and AD-related plasma biomarkers in older adults in the Connectomics in Brain Aging and Dementia study. A total of 180 participants (mean age=65.0y, 64.4%women, 55.56% Black) had valid 7-day wrist-worn accelerometer assessment and consensus cognitive diagnosis in 2016-2021. RAR measures were computed using nparACT R package including interdaily stability (IS), intradaily variability (IV), relative amplitude (RA), M10 (activity levels at the most active 10 hours), and L5 (activity levels at the least active 5 hours). Plasma biomarkers (Aβ42, Aβ40, phosphorylated tau [p-tau]181, p-tau217, p-tau231, glial fibrillary acidic protein [GFAP], and neurofilament light chain [NfL]) were measured. Logistic regression models showed that higher IS and higher M10 were associated with lower odds of MCI or dementia diagnosis (odds ratio [OR]=0.15, 95% confidence interval[CI]=0.03-0.82, p = 0.03; OR = 0.94, 95% CI = 0.90-0.98, p = 0.003, respectively) after adjusting for sociodemographic factors. No significant associations were found between RAR and Aβ or p-tau biomarkers. However, linear regression models showed that higher RA was significantly associated with lower GFAP levels (β=-164.3, p = 0.02). Findings suggest that more stable (IS) and robust (RA) rest-activity rhythms may serve as non-invasive markers of cognitive health in older adults. Circadian disruptions may be linked to neuroinflammatory processes more than amyloid or tau pathology, but warrant further investigations in longitudinal studies.

